# Baseline anxiety disorders are associated with progression of diabetic kidney disease in type 2 diabetes

**DOI:** 10.1080/0886022X.2022.2159431

**Published:** 2023-01-12

**Authors:** Bin Han, Ling Wang, Yueyue Zhang, Lijie Gu, Weijie Yuan, Wei Cao

**Affiliations:** a Department of Nephrology, Shanghai General Hospital, Shanghai Jiaotong University School of Medicine, Shanghai, China; b Department of Emergency Medicine, Affiliated Hospital of Jiaxing University, Jiaxing, China

**Keywords:** Diabetic kidney disease, type 2 diabetes, anxiety, depression, cohort study

## Abstract

**Background:**

Diabetic kidney disease (DKD) is a leading cause of kidney failure worldwide. Anxiety has been associated with disease progression in non-diabetes patients. We aimed to examine the prospective association between anxiety and progression of DKD in type 2 diabetes.

**Methods:**

We conducted a prospective cohort study of 2040 participants with type 2 diabetes at the Diabetes Center of Shanghai General Hospital between May 2017 and June 2020. Anxiety disorders at baseline were diagnosed by a structured clinical interview based on the 10th Revision of International Classification of Disease (ICD). Progression of DKD was identified as the transition from one urinary albumin excretion rate (AER) stage to the next or the development of kidney failure during the follow-up period.

**Results:**

At baseline, 403 (19.8%) had a diagnosis of anxiety disorders, of whom 107 (26.6%) also received a depression diagnosis. During a median follow-up time of 3.2 years, deterioration of the kidney status occurred in 340 (16.7%) individuals. After adjustment for potential confounders including depression or an anxiety × depression interaction term, anxiety disorders were independently related to an increased risk of progression of DKD (HR 1.539, 95% CI 1.130–2.095, *p* = 0.006; HR 1.536, 95% CI 1.111–2.122, *p* = 0.009, respectively).

**Conclusions:**

Anxiety disorders at baseline, independent of possible confounders, were associated with the progression of DKD in type 2 diabetes. Whether therapeutic interventions for anxiety reduce the risk needs to be investigated.

## Introduction

Diabetic kidney disease (DKD) is a common complication of type 2 diabetes and the leading cause of kidney failure worldwide [1]. It is clinically characterized by albuminuria, kidney function loss, or both [2]. The prevalences of both type 2 diabetes and DKD have been rising [3]. An emerging body of literature suggests that DKD tightly correlates with increased risks of cardiovascular events and mortality [4,5]. An understanding of modifiable risk factors related to the progression of DKD is therefore useful for establishing early interventions to prevent DKD progression and to improve clinical outcomes.

American Diabetes Association (ADA) recommends clinical screening for psychological distress, particularly anxiety and depression, among patients with diabetes. As with depression, anxiety is highly common among this patient population with 20% higher prevalence than general population [6]. A recent study by Weaver et al. displayed that high levels of anxiety were more prevalent than depression, especially during the first 2 years of type 2 diabetes [7]. Yet, the potential role of anxiety on diabetes has received much less attention than depression-diabetes comorbidity.

Diabetes complicated by anxiety has been linked with less optimal diabetes self-management [8], reduced quality of life [9], and high-cost health care use [10]. Importantly, anxiety in patients with diabetes is associated with worse glycaemic control [11], heightened inflammation, and increased risk of mortality [12,13]. Recently, several clinical research have shown a negative effect of anxiety on kidney function among patients with kidney transplantation or chronic kidney disease [14,15]. Presence of anxiety has been associated with proteinuria in non-diabetes patients [16,17]. A recent meta-analysis by Huang et al. revealed a pooled prevalence of anxiety disorders of 19% among patients with chronic kidney disease [18]. Additionally, anxiety contributes to the progression of numerous diseases, such as disability, mild cognitive impairment, carotid atherosclerosis, coronary artery disease, and prediabetes [19–23]. Currently, studies investigating the influence of anxiety on progression of DKD are lacking. Therefore, we studied a cohort of participants with type 2 diabetes to determine whether there was an association between anxiety and DKD progression during 3.2 year of follow-up.

## Materials and methods

### Study design and population

We conducted a prospective, observational cohort study of patients diagnosed with type 2 diabetes in the Diabetes Center of Shanghai General Hospital Affiliated to Shanghai Jiaotong University School of Medicine in Shanghai, China. Patients were consecutively recruited on a basis of their regular hospital visits in the period of 1 May 2017 and 31 June 2020 and were followed up from the baseline to the date of occurrence of a progression event, or 31 June 2021 if no event occurred. At baseline, attending physician ascertained diagnosis of type 2 diabetes on the basis of the Chinese diabetes care guidelines by Chinese Diabetes Society [24]. Exclusion criteria were as follows: (a) age <18 years; (b) having a diagnosis of kidney failure at baseline; (c) a life-threatening comorbidity such as cancer; (d) language/hearing disorders, or dementia; (e) missing clinical information; and, (f) decline to participate in the present investigation. The study protocol adhering to the Declaration of Helsinki was approved by the Ethics Committees of Shanghai General Hospital (2019KY014). The written informed consents were obtained from all individuals prior to participation.

### Demographic and clinical data

Clinical research coordinators used the electronic medical records of the hospital to collect demographic and clinical information, including sex, age, marital status, smoking, height and body weight, blood pressure, duration of diabetes, use of renin angiotensin system (RAS) blockers (i.e., angiotensin receptor blockers, angiotensin-converting enzyme blockers), and use of statins. Body mass index (BMI) was defined as the ratio of weight in kilograms and height in meters squared (kg/m^2^). Seated blood pressure was measured twice; the first measurement was conducted after a minimum of 10-min rest, and the second followed with a 2 min interval. Mean of the two measurements was calculated. Laboratory tests included serum concentrations of HbA_1c_, total cholesterol, HDL cholesterol, triglyceride, LDL cholesterol, and creatinine, as well as urinary albumin excretion rate (AER). Estimated glomerular filtration rate (eGFR) was calculated by the simplified Chinese Modification of Diet in Renal Disease (MDRD) equation. Hypertension was ascertained by the attending physician using the 10th Revision diagnosis code of the International Classification of Diseases (ICD-10).

### Anxiety disorders

At baseline, symptoms of anxiety and depression were screened using the Anxiety and Depression Scale (HADS), which is widely used in patients from non-psychiatric hospital clinics [25]. The HADS-Anxiety and HADS-Depression subscales each consist of 7 domains with a 4-point Likert scale [0–3], with a cutoff of ≥8 suggesting the presence of anxiety or depressive symptoms [25]. The scales were conducted by trained assistants in a separate room at 8:00 a.m. Anxiety disorders (panic disorder, generalized anxiety disorder, social phobia disorder) and depression were diagnosed using the Mini International Neuropsychiatric Interview, a widely used reliable and well-validated interview based on the ICD-10 [26]. The structured interview, which takes about 35 min to complete, was conducted by trained assistants who were blinded to the clinical data of the participants.

### Outcomes

Kidney status was evaluated on the basis of AER in at least two of three overnight or consecutive timed 24-h urine collections. Accordingly, participants were divided into four classifications: normal AER (<30 mg/24 h or <20 μg/min), microalbuminuria (≥30 but <300 mg/24 h or ≥20 but <200 µg/min), macroalbuminuria (≥300 mg/24 h or ≥200 μg/min), and kidney failure (having received a kidney transplantation or undergoing dialysis). DKD progression was identified as the transition from one AER stage to the next stage or the development of kidney failure (i.e., from normal AER at baseline to either microalbuminuria, macroalbuminuria, or kidney failure; or from microalbuminuria at baseline to either macroalbuminuria, or kidney failure; or from macroalbuminuria at baseline to kidney failure). Data on progression were derived from the medical records during the follow-up period.

### Statistical analysis

Descriptive statistics are reported as median (interquartile range (IQR)) or mean ± standard deviation (SD) for quantitative data, and percentages for categorical data. Normal distribution was examine using Kolmogorov-Smirnov test. Comparison between groups was conducted using Pearson’s *χ^2^* test for categorical data and Mann-Whitney *U* test for nonparametric distributions or independent samples *t* test for normally distributed data. To test the associations between diagnosed anxiety and the progression of DKD, we performed Kaplan–Meier analyses and Cox regression analyses. First, we performed univariate Cox regression analyses to investigate anxiety as a predictor for DKD progression. Next, we included age, sex, BMI, marital status, smoking, hypertension, diabetes duration, use of RAS blocker and statins, blood pressure, HbA_1c_, eGFR, total cholesterol, HDL cholesterol, triglyceride, LDL cholesterol, and an anxiety × depression interaction term in the multivariate Cox model (Model 1). Considering the possible interaction of depression with anxiety, we included an anxiety × depression interaction term in the multivariate Cox regression model. Finally, in order to examine independence from depression, we separately adjusted for depression (Model 2). We also tested interactions between the primary predictor and sex, age, hypertension, or diabetes duration. Results were presented as hazard ratios (HR) and 95% confidence intervals (CI). All analyses were conducted using SPSS 26.0 (Chicago, IL, USA). A two-tailed *p* < 0.05 was considered statistically significant.

## Results

### Baseline characteristics

A total of 2,433 type 2 diabetic individuals who visited our hospital, 393 were excluded from the current study because of cancer (*n* = 68), kidney failure at baseline (*n* = 103), hearing disorders (*n* = 17), language disorders (*n* = 13), dementia (*n* = 37), or missing clinical data (*n* = 22), or decline to participate in the current study (*n* = 129). In addition, 26 who were lost to follow-up were excluded. After these exclusions, 2040 eligible participants were included in the analyses (45.8% female, mean age 60.2 ± 6.9 years, mean duration of diabetes 10.5 ± 2.8 years, Table 1). No significant differences in regard to demographic and clinical data were observed between the eligible participants and those excluded (data not shown).

**Table 1.** Characteristics of participants by anxiety disorders.

Characteristics	All	No anxiety diagnosis	Anxiety diagnosis	*p* Value
*n*	2040 (−)	1637 (80.2)	403 (19.8)	
Female (%)	936 (45.8)	671 (41.0)	265 (65.8)	<0.001
Age (years)	60.2 ± 6.9	60.7 ± 6.5	58.1 ± 8.1	<0.001
BMI (kg/m^2^)	29.7 ± 14.9	29.1 ± 15.7	31.9 ± 11.2	0.001
Married/life partner (%)	1800 (88.2)	1451 (88.6)	349 (86.6)	0.255
Current smoking (%)	493 (24.2)	392 (23.9)	101 (25.1)	0.639
Hypertension (%)	945 (46.3)	746 (45.6)	199 (49.4)	0.170
Diabetes duration (years)	10.5 ± 2.8	10.0 ± 2.7	12.4 ± 2.2	<0.001
Total cholesterol (mmol/l)	5.06 ± 0.23	5.06 ± 0.23	5.05 ± 0.24	0.341
HDL cholesterol (mmol/l)	1.52 ± 0.49	1.52 ± 0.47	1.55 ± 0.56	0.177
Triglyceride (mmol/l)	1.88 ± 1.21	1.89 ± 1.28	1.85 ± 0.91	0.538
LDL cholesterol (mmol/l)	2.57 ± 0.85	2.56 ± 0.82	2.61 ± 0.96	0.289
HbA_1c_ (%)	7.7 ± 0.9	7.7 ± 0.8	7.8 ± 1.0	0.003
HbA_1c_ (mmol/mol)	59.8 ± 10.6	59.1 ± 10.2	62.8 ± 11.8	<0.001
AER (mg/24 h)	44 (23–91)	43 (25–89)	168 (56–224)	<0.001
eGFR (ml/min/1.73 m^2^)	79.6 ± 25.5	79.8 ± 25.3	78.7 ± 26.5	0.476
SBP (mmHg)	139.9 ± 8.5	138.9 ± 7.8	144.2 ± 9.5	<0.001
DBP (mmHg)	84.7 ± 7.0	84.6 ± 6.5	85.2 ± 8.5	0.149
RAS inhibitor (%)	851 (41.7)	692 (42.3)	159 (39.5)	0.304
Statin (%)	754 (37.0)	610 (37.3)	144 (35.7)	0.568
Diagnosed depression (%)	250 (12.3)	143 (8.7)	107 (26.6)	<0.001

Quantitative values are presented as mean ± SD, and qualitative values are presented as number of participants/percentage. BMI: body mass index; AER: albumin excretion rate; eGFR: estimated glomerular filtration rate; SBP: systolic blood pressure; DBP: diastolic blood pressure; RAS: renin angiotensin system; SD: standard deviation.

Of the 2040 individuals included in the current study, 403 (19.8%) had a diagnosis of anxiety disorders, of whom 202 (50.1) had a social phobia disorder, 127 (31.5%) had a panic disorder, 74 (18.4%) had a generalized anxiety disorder, and 107 (26.6%) also received a depression diagnosis. Participants with anxiety disorders were more likely to be female, younger, had a longer diabetes duration, and had higher levels of BMI, SBP, and AER, compared with those without anxiety disorders. A statistically significant but clinically tiny difference in levels of HbA_1c_ was observed in those with and without anxiety disorders (7.8 vs. 7.7%, *p* = 0.003; 59.1 ± 10.2 vs. 62.8 ± 11.8 mmol/mol, *p* < 0.001, respectively). There were no significant differences in regard to other laboratory parameters, prevalence of hypertension, or use of renin-angiotensin system blocker (Table 1).

### Association between anxiety disorders and progression of DKD

**Figure 1.**
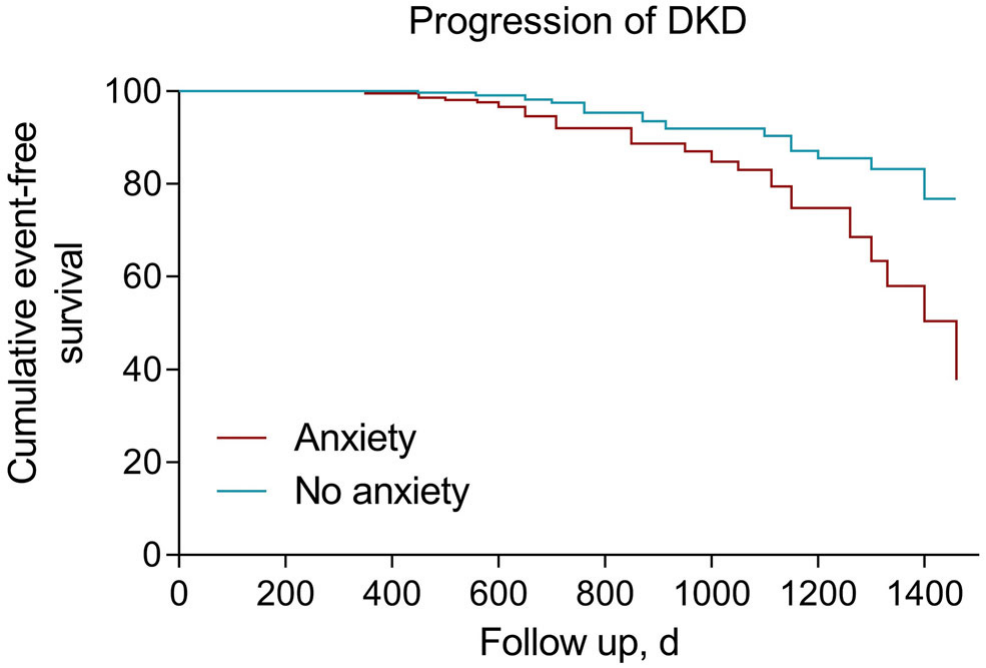
Cumulative event-free survival (Kaplan–Meyer curves) for progression of DKD. Significant difference was shown in estimated mean time free of DKD progression by anxiety status (log rank *p* < 0.001). DKD: diabetic kidney disease.

Over the median follow-up time of 3.2 (2.5–3.5) years, deterioration of the kidney status occurred in 340 (16.7%) individuals, ranging from 14.7% in participants without anxiety to 24.8% in those with anxiety (*p* < 0.001). The cumulative incidence of the progression by anxiety status is depicted in Figure 1 (*p* < 0.001 by log rank test). The progressors tended to be older, less likely to be female, had a longer diabetes duration, higher prevalence of hypertension, less use of RAS blocker and statin, higher levels of total cholesterol, when compared to the non-progressors. Of importance, a higher proportion of participants with deteriorating kidney status had a diagnosed anxiety or depression (28.8 vs. 17.9%, *p* < 0.001; 17.9 vs. 11.1%, *p* < 0.001, respectively, Table 2)

**Table 2.** Characteristics of participants by progression of diabetic nephropathy.

Characteristics	Non-progressors	Progressors	*p* Value
*n*	1700 (83.3%)	340 (16.7%)	
Female (%)	816 (48.0)	120 (35.3)	<0.001
Age (years)	60.0 ± 7.0	61.1 ± 7.2	0.006
BMI (kg/m^2^)	29.6 ± 14.4	30.0 ± 17.6	0.668
Married/life partner (%)	1502 (88.4)	295 (86.8)	0.409
Current smoking (%)	405 (23.8)	88 (25.9)	0.418
Hypertension (%)	765 (45.0)	180 (52.9)	0.007
Diabetes duration (years)	10.4 ± 2.7	10.8 ± 3.4	0.008
Follow-up time (years)	3.0 (2.1–3.2)	2.1 (0.9–3.2)	<0.001
Total cholesterol (mmol/l)	5.05 ± 0.23	5.09 ± 0.24	0.004
HDL cholesterol (mmol/l)	1.53 ± 0.49	1.51 ± 0.47	0.678
Triglyceride (mmol/l)	1.89 ± 1.26	1.87 ± 0.96	0.859
LDL cholesterol (mmol/l)	2.57 ± 0.86	2.58 ± 0.80	0.788
HbA_1c_ (%)	7.6 ± 1.0	7.7 ± 0.92	0.250
HbA_1c_ (mmol/mol)	59.7 ± 10.7	60.5 ± 10.1	0.250
eGFR (ml/min/1.73 m^2^)	79.9 ± 25.9	77.8 ± 23.5	0.168
SBP (mmHg)	139.3 ± 7.5	142.9 ± 11.3	<0.001
DBP (mmHg)	84.8 ± 7.0	84.7 ± 7.0	0.870
RAS inhibitor (%)	733 (43.1)	119 (35.0)	0.006
Statin (%)	648 (38.1)	106 (31.2)	0.015
Anxiety disorders (%)	305 (17.9)	98 (28.8)	<0.001
Depression (%)	189 (11.1)	61 (17.9)	<0.001

Quantitative values are presented as mean ± SD or median (IQR), and qualitative values are presented as number of participants/percentage. BMI: body mass index; eGFR: estimated glomerular filtration rate; SBP: systolic blood pressure; DBP: diastolic blood pressure; RAS: renin angiotensin system; SD: standard deviation.

Using Cox proportional hazards models, diagnosed anxiety disorders were significantly associated with an increased risk of the progression of kidney status (Table 3). In univariate Cox regression analyses, participants with anxiety disorders had a 1.65-fold higher risk of kidney decline (95% CI 1.278–2.136, *p* < 0.001). After adjusting for possible confounding factors (age, sex, BMI, marital status, smoking, hypertension, diabetes duration, use of RAS blocker and statins, HbA_1c_, eGFR, total cholesterol, HDL cholesterol, triglyceride, LDL cholesterol, and an anxiety × depression interaction term), anxiety disorders had a strong positive association with the odds of DKD progression (HR 1.536, 95% CI 1.111–2.122, *p* = 0.009). The association remained robust in the model adjusted additionally for depression (HR 1.539, 95% CI 1.130–2.095, *p* = 0.006). Moreover, interactions between anxiety status and sex, age, hypertension, or diabetes duration, were not significant (All *p* > 0.05).

**Table 3.** Association between anxiety and progression of diabetic nephropathy in type 2 diabetes.

	HR	95% CI	*p* Value
Univariate model	1.652	1.278–2.136	<0.001
Multivariate-adjusted model			
Model 1*	1.553	1.122–2.150	0.008
Model 2^†^	1.539	1.130–2.095	0.006

*Model 1 is adjusted for age, sex, BMI, marital status, smoking, hypertension, duration of diabetes, use of renin-angiotensin system blocker and statin, HbA_1c_, eGFR, total cholesterol, HDL cholesterol, triglyceride, LDL cholesterol, blood pressure, and an anxiety × depression interaction term. ^†^Model 2 is adjusted additionally for depression. BMI: body mass index; eGFR: estimated glomerular filtration rate; HR: hazard ratios; CI: confidence intervals.

## Discussion

To our best knowledge, this study is the first to examine the association between anxiety and the progression of DKD. We found that independent of traditional related factors for DKD, including age, sex, smoking, duration of diabetes, blood pressure status, glycemic control, hypertension, dyslipidemia, RAS blockers and statins use, as well as depression, diagnosed anxiety disorders at baseline were found to increase the risk of progression of DKD. The observation is in line with a recent study, which has shown that anxiety/depression is related to an increased risk of incidence of chronic kidney disease among people with diabetes [27]. Similarly, Castellano-Guerrero et al. found that anxious symptomatology independently correlates with microvascular and macrovascular complications in individuals with diabetes [28].

Common risk factors of anxiety in patients with DN remain unknown. In the present study, we found that participants with anxiety disorders were more likely to be female, younger, had a longer diabetes duration, and had higher levels of BMI, SBP, and AER, compared with those without anxiety disorders. Prospective studies with large sample size are needed.

As mentioned earlier, a large body of investigations demonstrated an important association of presence of anxiety with disease progression. In a community-based observational study exploring the influence of anxiety on the progression of disability, at the end of 6-month follow-up, baseline anxiety was a significant predictor for the progression of disability in older women [19]. In a recent review and meta-analysis of eleven investigations, anxiety was linked with an increased risk of the progression to dementia among individuals with mild cognitive impairment [20]. Paterniti et al. found that high levels of anxiety contributes to the evolution of carotid atherosclerosis [21]. In a longitudinal cohort of 247 participants with prediabetes, anxiety symptoms at baseline were correlated with a 3-fold increased risk of the progression to type 2 diabetes [22].

The mechanisms underlying the link between anxiety and poor outcomes of diabetes may be associated with biological and behavioral factors. Anxiety in patients with diabetes has been associated with worse glycemic control [11], and thus increase the risk of diabetic complications. Additionally, a recent review by Groot et al. demonstrated that anxiety is linked with poor diabetes self-management in people with diabetes [8]. The less optimal diabetes self-care practices may be an indication of inability to administrate the complexities of the disease or reduced attention to one’s physical health, and subsequently favor the development and progression of diabetes-related complications. However, the exact mechanisms connecting anxiety to progression of DKD remains to be elucidated. It is well-known that oxidative stress and activation of RAS are implicated in the pathophysiology of DKD [29,30]. Anxiety disorders have been shown to be associated with oxidative stress [31,32]. A recent systematic review of animal studies demonstrated that anxiety state comprehends a cascade of neuroendocrine processes, particularly activation of RAS [33]. Thus, it is conceivable that anxiety contributes to the progression of DKD, at least in part, via oxidative stress and activation of RAS. Another possible explanation is its involvement in inflammation. Increasing evidences emphasized the key role of inflammation in the development and progression of DKD [34–36]. Of importance, anxiety in patients with diabetes is associated with heightened inflammation [13]. Therefore, anxiety may drive DKD through enhancing inflammatory response.

It is possible that other psychological variables, depression in particular, could interact with anxiety and thus could affect the relationship between anxiety and the progression of DKD. Determining the strength of each psychological factors and differentiating their complex interactions is, however, beyond what the present study design permits. In our study, depression and its interaction term with anxiety was included in the multivariate Cox regression model as covariates. We observed that the interaction term was not significant, and that anxiety remained to be a strong predictor of DKD progression. Moreover, the association remained robust when depression was entered into the multivariate model. Collectively, the prognostic value of diagnosed anxiety seems to be independent of depression status.

Our results need to be considered in view of the limitations of our study. First, all participants were of Chinese descent only and were enrolled from a single diabetes unit, thereby limiting generalization of the findings of this study. Second, individuals who refused to give written informed consent and individuals with kidney failure at baseline, excluded from this cohort study. We tried to diminished the potential bias by enrolling participants consecutively. Third, although we adjusted for a large number of potential confounding factors, the potential for residual confounding remains, as we had limited information on other unmeasured confounders related to the risk of DKD. Fourth, owing to lack of data on hospitalization rates and incidence of acute kidney injury, both of which are prognostic risk factors for DKD, we were not able to include them in our analyses. Fifth, the psychiatric diagnosis was not confirmed by a psychiatrist. Finally, the present study did not account for medication use of antianxietic/antidepressant, which is known to correlate with poor glycemic control and diabetes-related complications [37,38]. Despite the limitations, our study has substantial strengths. First, diagnosed anxiety/depression was ascertained by the gold standard clinical interviews based on the 10th Revision of ICD. Second, the longitudinal study design enables us to test the causal association of anxiety with progression of DKD. Finally, our study included data on a comprehensive range of potential confounding factors and a reliable source for diabetes-related data from a relatively large population.

In conclusion, our study demonstrates that anxiety disorders at baseline, independent of traditional risks factors and depression, are associated with an increased risk of the progression of DKD, among individuals with type 2 diabetes. Whether recognizing individuals with anxiety and targeting therapeutic interventions could guard against the progression of DKD should be investigated.

## Data Availability

The datasets during and/or analyzed during the present study are available from the corresponding author on reasonable request.
